# Scoliosis corrective force estimation from the implanted rod deformation using 3D-FEM analysis

**DOI:** 10.1186/1748-7161-10-S2-S2

**Published:** 2015-02-11

**Authors:** Yuichiro Abe, Manabu Ito, Kuniyoshi Abumi, Hideki Sudo, Remel Salmingo, Shigeru Tadano

**Affiliations:** 1 Department of Orthopaedic Surgery, Wajokai Eniwa Hospital, Eniwa, Hokkaido, Japan; 2 Department of Orthopaedic Surgery, Hokkaido Medical Center, Sapporo, Hokkaido, Japan; 3 Department of Orthopaedic Surgery, Sapporo Orthopaedic Hospital, Sapporo, Hokkaido, Japan; 4 Department of Orthopaedic Surgery, Hokkaido University, Sapporo, Hokkaido, Japan; 5 Division of Human Mechanical Systems and Design, Faculty of Engineering, Hokkaido University, Sapporo, Hokkaido, Japan

## Abstract

**Background:**

Improvement of material property in spinal instrumentation has brought better deformity correction in scoliosis surgery in recent years. The increase of mechanical strength in instruments directly means the increase of force, which acts on bone-implant interface during scoliosis surgery. However, the actual correction force during the correction maneuver and safety margin of pull out force on each screw were not well known. In the present study, estimated corrective forces and pull out forces were analyzed using a novel method based on Finite Element Analysis (FEA).

**Methods:**

Twenty adolescent idiopathic scoliosis patients (1 boy and 19 girls) who underwent reconstructive scoliosis surgery between June 2009 and Jun 2011 were included in this study. Scoliosis correction was performed with 6mm diameter titanium rod (Ti6Al7Nb) using the simultaneous double rod rotation technique (SDRRT) in all cases. The pre-maneuver and post-maneuver rod geometry was collected from intraoperative tracing and postoperative 3D-CT images, and 3D-FEA was performed with ANSYS. Cobb angle of major curve, correction rate and thoracic kyphosis were measured on X-ray images.

**Results:**

Average age at surgery was 14.8, and average fusion length was 8.9 segments. Major curve was corrected from 63.1 to 18.1 degrees in average and correction rate was 71.4%. Rod geometry showed significant change on the concave side. Curvature of the rod on concave and convex sides decreased from 33.6 to 17.8 degrees, and from 25.9 to 23.8 degrees, respectively. Estimated pull out forces at apical vertebrae were 160.0N in the concave side screw and 35.6N in the convex side screw. Estimated push in force at LIV and UIV were 305.1N in the concave side screw and 86.4N in the convex side screw.

**Conclusions:**

Corrective force during scoliosis surgery was demonstrated to be about four times greater in the concave side than in convex side. Averaged pull out and push in force fell below previously reported safety margin. Therefore, the SDRRT maneuver was safe for correcting moderate magnitude curves. To prevent implant breakage or pedicle fracture during the maneuver in a severe curve correction, mobilization of spinal segment by releasing soft tissue or facet joint could be more important than using a stronger correction maneuver with a rigid implant.

## Background

Development of novel correction maneuvers and improvement of material property in spinal instrumentation have resulted in better deformity correction in scoliosis surgery in recent years [1,2]. Rod rotation maneuver and direct vertebral rotation technique using thoracic pedicle screw have provided better scoliosis correction, and use of rigid instruments such as cobalt-chromium alloy rod have also accelerated these trends of aggressive correction. The use of these aggressive correction maneuver could induce the increase of force exerted on bone-implant interface during scoliosis surgery. There were many biomechanical studies about holding strength of spinal instruments [3-6]. As regards corrective force exerted on the spinal instruments, several biomechanical models were developed to simulate scoliosis correction in-vitro [7-9]. However, there were only few reports which estimated the actual corrective force during correction maneuver in vivo [10,11]. We have developed a novel method for estimating corrective force acting on the spine by investigating the geometrical change of implanted rod in scoliosis surgery [12,13]. In the present study, estimated corrective forces and pull out forces exerted on the pedicle screw were analyzed using a novel method based on finite element analysis.

## Methods

**Figure 1:**
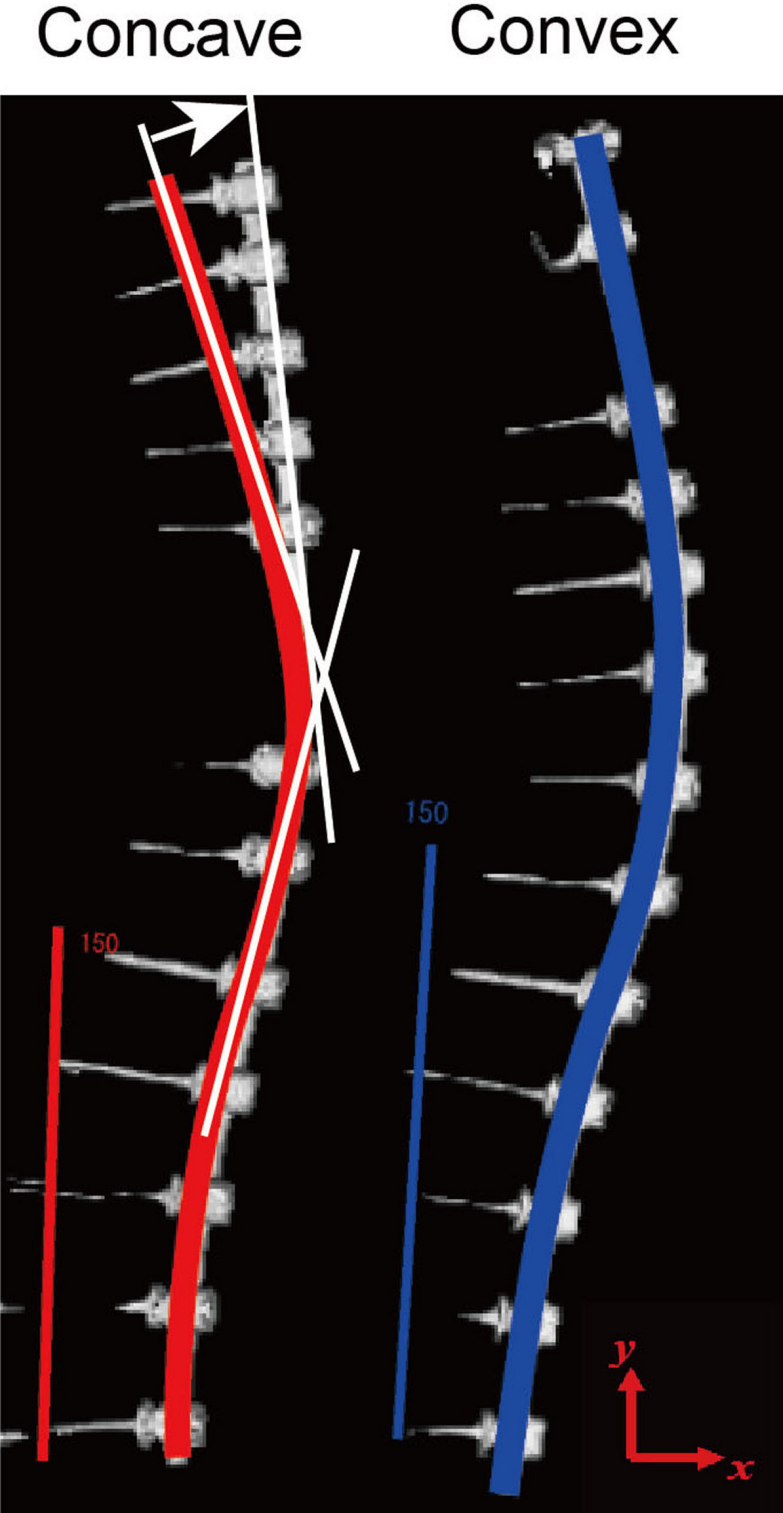
**Collection of rod geometry.** The rod geometry was collected from intraoperative tracing and postoperative 3D-CT images. Rod contour decreased in postoperative 3D-CT (arrow).

Twenty adolescent idiopathic scoliosis patients (1 boy and 19 girls) who underwent reconstructive scoliosis surgery between June 2009 and June 2011 were included in this study. All 20 patients had Type 1 curve. 12 patients had Type 1A curve, 4 had Type 1B curve and 4 fad Type 1C curve. Curve magnitude of major thoracic curve was 63.1 ± 6.8 degrees in average (range: 53 – 74). Scoliosis correction was performed with 6mm diameter titanium rod (Ti6Al7Nb) using the simultaneous double rod rotation technique (SDRRT) in all cases [14]. The pre-maneuver and post-maneuver rod geometry was collected from intraoperative tracing and postoperative 3D-CT images, and the changes in the rod curvature were measured (Fig.1). The initial geometry of implant rod was measured from the actual rod used before surgery. High-resolution photograph was taken before insert the rod, and liner model with 6 mm diameter was created using CAD software Solidworks 2010 (Dassault Systemes, Massachusetts, USA). The implant rod geometry after surgery was obtained a week after the surgical operation using Aquilion 64 CT scanner (Toshiba Medical Systems Corporation, Tochigi, Japan). The slice thickness was 0.5mm. The images were imported into Solidworks 2010 to measure the 3D geometry and deformation. Cobb angle of major curve, correction rate and thoracic kyphosis were measured on X-ray images. The study was approved by the Ethical Committee for Clinical Research at Wajokai Eniwa Hosiptal (approval date: April 1, 2011).

## FEM

3D-CAD model of rod geometry was generated and Finite Element Analysis (FEA) was performed using ANSYS 11.0 according to the previously developed method reported by Salmingo et al [12,13]. Material properties of this rod are Young's Modulus (E), yield stress (σY), yield strain (εY) and hardening coefficient (H) equal to105 GPa, 900 MPa, 8.57×10−3 and 2.41 GPa, respectively.

A zero force *F_i_* (*i* = no. of screws) was applied initially to the corresponding location of each screw on the rod geometry before surgery. An elasto-plastic deformation analysis was performed. The displacement vector  required to attain the location of each screw after surgery was used in the iterative process. The applied forces to the rod before surgery were replaced by adding the value of the displacement vector . The whole process was iterated until the displacement vector  was minimized or the evaluating function was met. The evaluating function is defined as the sum of the squares of displacement vector  expressed in Eq. (1). The force iteration process and the finite element deformation analysis was stopped when the evaluating function was less than µ (where µ = 0.5). The rod model was deformed the same rod geometry after surgery because the displacement vector  was minimized or close to zero.(1)

Push out or push in forces exerted on each screw on the bilateral side were estimated using FEA. The calculated forces represent also to the pullout and push-in forces acting at the vertebrae of the scoliosis patient since the implant is directly connected to the vertebra. The magnitudes of pullout and push-in forces were also estimated from the applied forces. The pullout or push-in force was defined as the pulling or pushing force acting normal to the implant curvature at the local coordinate plane (y-x plane since the rod is bent at one plane), Fig. 1. The pullout or push-in force was computed using the reaction force (i.e. opposite force acting at the spine) of the computed corrective force that deformed the rod during scoliosis surgery. The rod geometry was used to define the direction of the pullout or push-in force because its curvature constitutes the spine curvature after the surgical treatment of scoliosis. The rod geometry was approximated by quintic polynomial function using the previous method [13]. A tangent angle β that is orthogonal to the pullout or push-in force axis was computed by evaluating the derivative of quintic polynomial function evaluated for location of each screw. The reaction force vector was resolved into component (Reaction force x Cosβ), equal to the magnitude of pullout or push-in force acting at the corresponding fixation level.

## Results

### 1. Measurement of radiological parameters

**Figure 2:**
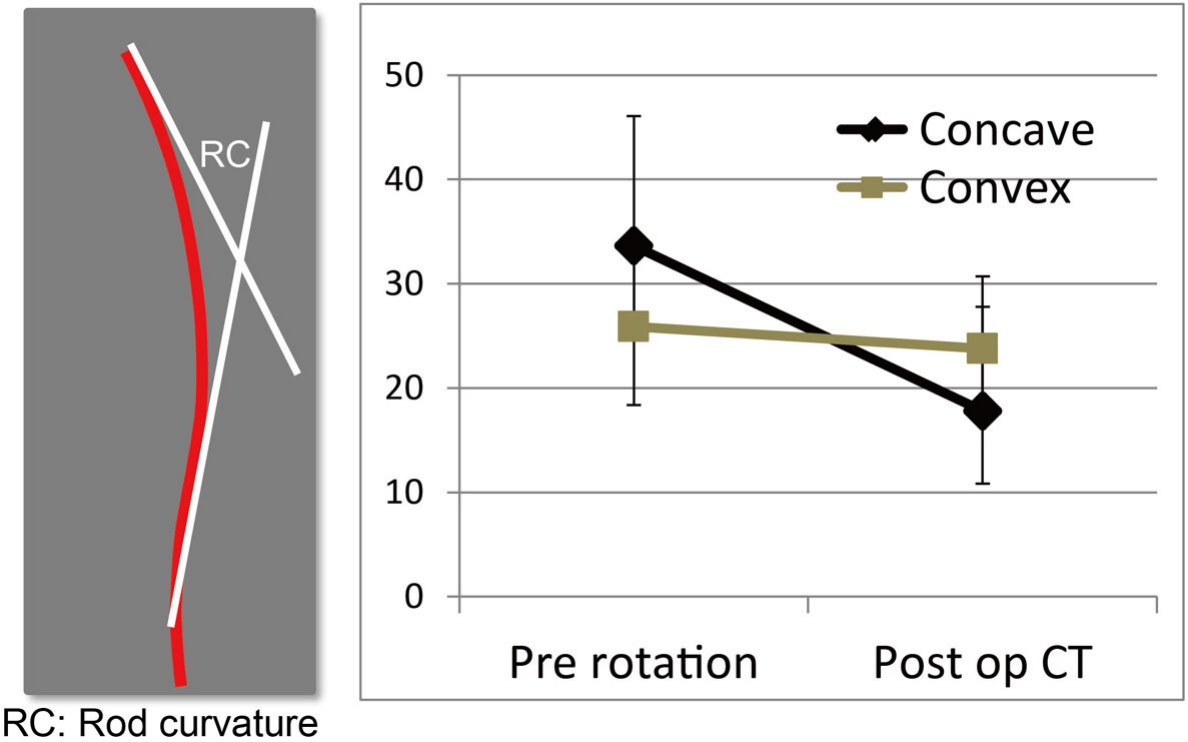
**Change in rod geometry.** Rod geometry showed significant change on the concave side compared with convex side.

Average age at surgery was 14.8 and average fusion length was 8.9 segments. Implant density in the current patients were 1.79 (range: 1.6 -2.0). Major curve was corrected from 63.1 to 18.1 degrees in average and correction rate was 71.4%. Rod geometry showed significant change on the concave side. Curvature of the rod on the concave and convex sides decreased from 33.6 to 17.8 degrees, and from 25.9 to 23.8 degrees, respectively (Fig. 2).

### 2. FEM

**Figure 3:**
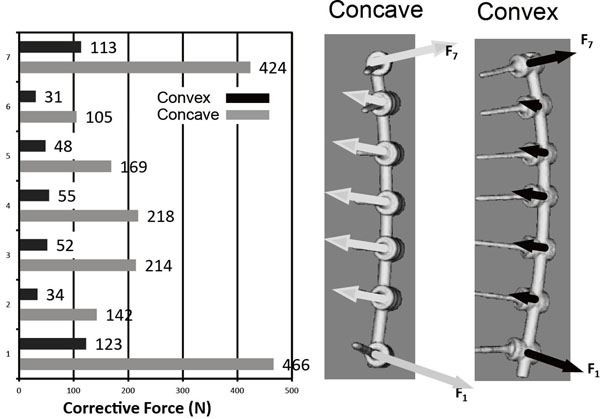
**Estimated forces on screws in representative case.** Estimated corrective force was four times greater in the concave side than in convex side. Pull out force was greatest in the apical level (218N) with smooth transition from one to another and pushing-in forces were high on pedicle screws at the both ends of rod.

**Figure 4:**
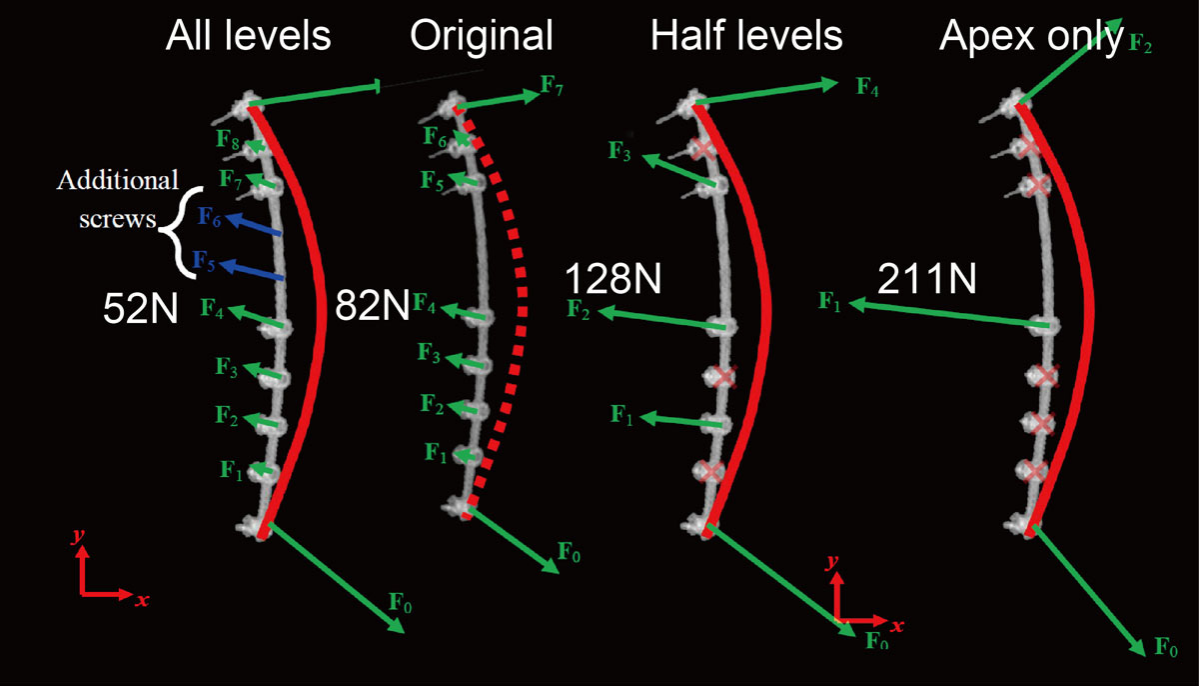
**Simulation model of different screw density.** As the number of screws decreased, the pull out force exerted on the apical screw significantly increased.

**Figure 5:**
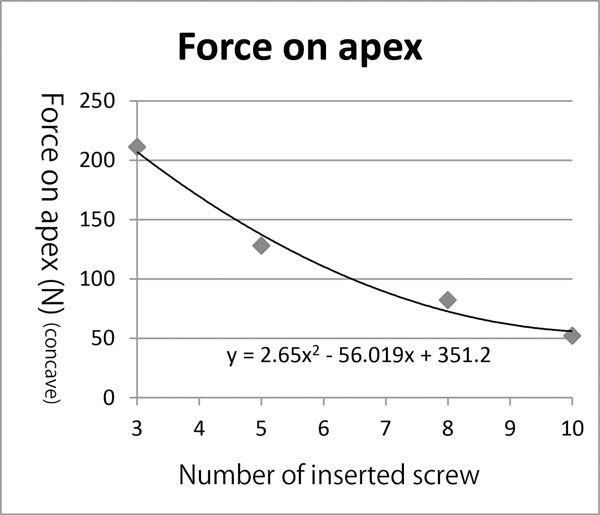
**Relationship between screw number and forces on screws.** The pull out force exerted on the apical screw decreased in an exponential manner when screw number increased. Pull out force at the apical level on the concave side was 4.1 fold greater in the apex only model (screw number = 3) than all inserted model (screw number =10).

Estimated pull out forces at apical vertebrae were 160.0 ± 81.1N in the concave side screw and 35.6 ± 15.1N in the convex side screw. Estimated push in force at LIV and UIV were 305.1 ± 131.6N in the concave side screw and 86.4 ± 44.0N in the convex side screw. Forces exerted on each screw, estimated using FEA in the representative case are shown in figure 3. We also evaluated the relationship between screw density and forces on screws (Fig. 4). In the presented case, as the number of screws decreased, the pull out force significantly increased. Pull out force at the apical level on the concave side was 2.5 fold greater in the half level inserted model compared to the all level inserted model, and 4.1 fold greater in the apex only model (Fig. 5).

## Discussion

Essentially, corrective force cannot be exerted on the spinal implant beyond the limit of anchor holding strength during scoliosis surgery. If corrective force reaches the limit of anchor strength, implant breakage or bony fracture reported as “screw plowing” could occur [9]. According to the results of the present study, averaged pull out and push in force exerted on each screw during SDRRT fell below previously reported safety margin [6], and therefore this maneuver was demonstrated to be safe for correcting a moderate magnitude curve. To prevent implant breakage or pedicle fracture during maneuver in more severe curve correction, destabilization of spinal segment by releasing soft tissue or facet joint could be more important rather than the use of excessive correction maneuver with rigid implant.

Less screws tend to be used in scoliosis surgery for economical reasons in recent years, and correction rates have been reported as not significantly different between the less density (1.0-1.25) group and the high density (1.75-2.0) group [15]. Reduction of the implant density, however, also means the load exerted on each anchor is increased. Our simulation study showed that pull out force exerted on the screw in the apical vertebra increased 2.5 fold when screw density decreased from 2.0 to 1.0. To solve these problems, we can use the current simulation technology to estimate in-vivo forces on each spinal implant, to find out the safety margin of implant forces and to determine the best settings of anchors and correction maneuvers for scoliosis correction.

## Conclusions

Corrective force during correction maneuver in scoliosis surgery and pull out forces acting on each pedicle screw were analyzed using a novel method based on Finite Element Analysis. Computational simulation based on in-vivo data makes difficult scoliosis surgery scientifically validated, which can improve safety and effectiveness of deformity correction.

This is the extended abstract of IRSSD 2014 program book [16].

## Consent to participate

Consent to publish for the article’s content and images provided as well as consent to participate was obtained from the patients and patient’s parents for this study

## Competing interests

The authors declare that they have no competing interests.

## Authors' contributions

Yuichiro Abe has made contributions to conception and design, and has been involved in drafting the manuscript. Remel Salmingo, Hideki Sudo and Kuniyoshi Abumi have made contributions to acquisition and analysis of data. Manabu Ito and Shigeru Tadano have made contribution to study design, revising manuscript and have given final approval of the version to be published.

## Declarations

Publication charges for this collection were funded by IRSSD 2014 Sapporo.

This article has been published as part of *Scoliosis* Volume 10 Supplement 2, 2015: Research into Spinal Deformities 9: Short Papers from the IRSSD 2014 Meeting. The full contents of the supplement are available online at http://www.scoliosisjournal.com/supplements/10/S2.

## References

[B1] Lee SM Suk SI Chung ER Direct vertebral rotation: a new technique of three-dimensional deformity correction with segmental pedicle screw fixation in adolescent idiopathic scoliosis Spine (Phila Pa 1976) 2004 29 343 349 10.1097/01.BRS.0000109991.88149.19 14752361

[B2] Suk SI Kim JH Kim SS Lim DJ Pedicle screw instrumentation in adolescent idiopathic scoliosis (AIS) Eur Spine J 2012 21 13 22 10.1007/s00586-011-1986-0 21874625 PMC3252448

[B3] Butler TEJ Asher MA Jayaraman G Nunley PD Robinson RG The strength and stiffness of thoracic implant anchors in osteoporotic spines Spine (Phila Pa 1976) 1994 19 1956 1962 10.1097/00007632-199409000-00016 7997929

[B4] GaGayet LE Pries P Hamcha H Clarac JP Texereau J Biomechanical study and digital modeling of traction resistance in posterior thoracic implants Spine (Phila Pa 1976) 2002 27 707 714 10.1097/00007632-200204010-00007 11923663

[B5] Murakami H Yamazaki K Attallah-Wasif ES Tsai KJ Shimamura T Hutton WC A biomechanical study of 3 different types of sublaminar wire used for constructs in the thoracic spine J Spinal Disord Tech 2006 19 442 446 10.1097/00024720-200608000-00012 16891981

[B6] Pfeiffer M Gilbertson LG Goel VK Griss P Keller JC Ryken TC Effect of specimen fixation method on pullout tests of pedicle screws Spine (Phila Pa 1976) 1996 21 1037 1044 10.1097/00007632-199605010-00009 8724087

[B7] Aubin CE Labelle H Chevrefils C Desroches G Clin J Eng AB Preoperative planning simulator for spinal deformity surgeries Spine (Phila Pa 1976) 2008 33 2143 2152 10.1097/BRS.0b013e31817bd89f 18794755

[B8] Aubin CE Petit Y Stokes IA Poulin F Gardner-Morse M Labelle H Biomechanical modeling of posterior instrumentation of the scoliotic spine Comput Methods Biomech Biomed Engin 2003 6 27 32 10.1080/1025584031000072237 12623435

[B9] Wagner MR Flores JB Sanpera I Herrera-Soto J Aortic abutment after direct vertebral rotation: plowing of pedicle screws Spine (Phila Pa 1976) 2011 36 243 247 10.1097/BRS.0b013e31820107d0 21248592

[B10] Wang X Aubin CE Crandall D Labelle H Biomechanical comparison of force levels in spinal instrumentation using monoaxial versus multi degree of freedom postloading pedicle screws Spine (Phila Pa 1976) 2011 36 E95 E104 10.1097/BRS.0b013e3181f07cca 21228695

[B11] Wang X Aubin CE Crandall D Labelle H Biomechanical modeling and analysis of a direct incremental segmental translation system for the instrumentation of scoliotic deformities Clin Biomech (Bristol, Avon) 2011 26 548 555 10.1016/j.clinbiomech.2011.01.011 21334124

[B12] Salmingo R Tadano S Fujisaki K Abe Y Ito M Corrective force analysis for scoliosis from implant rod deformation Clin Biomech (Bristol, Avon) 2012 27 545 550 10.1016/j.clinbiomech.2012.01.004 22321374

[B13] Salmingo RA Tadano S Fujisaki K Abe Y Ito M Relationship of forces acting on implant rods and degree of scoliosis correction Clin Biomech (Bristol, Avon) 2012 10.1016/j.clinbiomech.2012.12.001 23273729

[B14] Ito M Abumi K Kotani Y Takahata M Sudo H Hojo Y Simultaneous double-rod rotation technique in posterior instrumentation surgery for correction of adolescent idiopathic scoliosis J Neurosurg Spine 2010 12 293 300 10.3171/2009.9.SPINE09377 20192630

[B15] Quan GM Gibson MJ Correction of main thoracic adolescent idiopathic scoliosis using pedicle screw instrumentation: does higher implant density improve correction? Spine (Phila Pa 1976) 2010 35 562 567 10.1097/BRS.0b013e3181b4af34 20118842

[B16] Yuichiro Abe Scoliosis corrective force estimation from the implanted rod deformation using 3D-FEM analysis Scoliosis 2015 10 Suppl 1 O23 10.1186/1748-7161-10-S2-S2 PMC4331738 25810754

